# Two or more dexamethasone intravitreal implants in treatment-naïve patients with macular edema due to retinal vein occlusion: subgroup analysis of a retrospective chart review study

**DOI:** 10.1186/s12886-015-0106-z

**Published:** 2015-09-04

**Authors:** Pravin U. Dugel, Antonio Capone, Michael A. Singer, Richard F. Dreyer, David G. Dodwell, Daniel B. Roth, Rui Shi, John G. Walt, Lanita C. Scott, David A. Hollander, for the SHASTA Study Group

**Affiliations:** Retinal Consultants of Arizona, 1101 E. Missouri Avenue, P.O. Box 32530, Phoenix, AZ 85014-2709 USA; Associated Retinal Consultants, Novi, MI USA; Medical Center Ophthalmology Associates, San Antonio, TX USA; Retina Northwest PC, Portland, OR USA; Illinois Retina Center, Springfield, IL USA; Retina-Vitreous Center, Rutgers-Robert Wood Johnson Medical School, New Brunswick, NJ USA; Allergan, Inc., Irvine, CA USA

## Abstract

**Background:**

Dexamethasone intravitreal implant (DEX implant) is a biodegradable, sustained-release implant that releases dexamethasone for up to 6 months. We evaluated the efficacy and safety of DEX implant in the treatment of macular edema secondary to retinal vein occlusion (RVO) in treatment-naïve patients.

**Methods:**

A multicenter, retrospective, open-label chart review study investigated the efficacy and safety of DEX implant treatment in 289 patients with macular edema secondary to branch or central RVO (BRVO, CRVO) who received ≥2 treatments with DEX implant in the study eye. Concomitant adjunctive RVO treatments were permitted. Data collected from the time of the first implant (baseline) to 3–6 months after the last implant included best-corrected visual acuity (BCVA) and central retinal thickness measured with optical coherence tomography. In this subgroup analysis, we evaluated outcomes in patients who had received no previous treatment for RVO complications.

**Results:**

Thirty-nine patients were treatment-naïve at the time of their first DEX implant (18 BRVO, 21 CRVO). Before the initial DEX implant, the mean duration of macular edema in treatment-naïve patients was 4.9 months, mean central retinal thickness was 550 μm, and mean Early Treatment Diabetic Retinopathy Study BCVA was 8.5 lines (20/125 Snellen). Treatment-naïve patients received a mean of 2.9 implants, either as monotherapy (*n* = 12) or with adjunctive RVO treatments (*n* = 27). The mean interval between implants was 177 days. After the first through sixth implants, mean changes from baseline BCVA ranged from +3.0 − +8.0 lines, and mean decreases from baseline central retinal thickness ranged from 241–459 μm. BCVA improved in both BRVO and CRVO and in both phakic and pseudophakic eyes. Overall, 83.8 % of treatment-naïve patients gained ≥2 lines in BCVA, 70.3 % gained ≥3 lines in BCVA, and 56.4 % achieved central retinal thickness ≤250 μm. The most common adverse event was increased intraocular pressure. Fifteen treatment-naïve patients had intraocular pressure ≥25 mm Hg; none required laser or incisional glaucoma surgery.

**Conclusion:**

Treatment with 2 or more DEX implants had a favorable safety profile and improved visual acuity and anatomic outcomes when used, either alone or with adjunctive RVO therapy, as initial treatment for RVO-associated macular edema.

**Trial registration:**

ClinicalTrials.gov NCT01411696, registered on August 5, 2011.

## Background

Macular edema associated with retinal vein occlusion (RVO) is a common cause of vision loss [1]. Intravitreal corticosteroids and antagonists of vascular endothelial growth factor (VEGF) have become first-line treatment for macular edema associated with RVO [2]. Intravitreal injection of the corticosteroid triamcinolone has been shown to result in decreased aqueous humor concentrations of multiple proinflammatory and angiogenic cytokines, including VEGF, that are associated with vascular leakage and macular edema [3]. Dexamethasone is a potent corticosteroid with well-established anti-inflammatory [4] and anti-VEGF [5, 6] effects. Dexamethasone intravitreal implant (DEX implant), the first approved medical treatment for macular edema following RVO, provides sustained release of dexamethasone for up to 6 months [7].

In 1-year, phase 3 trials for regulatory approval, DEX implant was shown to effectively reduce macular edema and improve visual acuity following both branch and central RVO (BRVO, CRVO) [8, 9]. In these trials, a second DEX implant was administered when needed at 6 months after the initial DEX implant [9]. Peak improvement in best-corrected visual acuity (BCVA) was observed at 2 months after each implant [8, 9]. In clinical practice, DEX implant may be used in combination with anti-VEGF and laser treatments following RVO. The optimal reinjection interval for DEX implant when used alone or in combination with other treatments has not been determined.

The SHASTA study was a multicenter chart review of safety and efficacy outcomes in 289 patients with macular edema associated with BRVO or CRVO who were treated with at least 2 DEX implants, with or without adjunctive RVO treatments, and were followed for 3 to 6 months after the last DEX implant [10]. In the total patient population, the results showed similar mean improvements in BCVA and central retinal thickness by optical coherence tomography (OCT) after each of the first through sixth DEX implant injections [10]. However, the patient population was diverse with respect to the duration of macular edema associated with RVO, previous treatment for RVO, and use of adjunctive treatment with DEX implant. Outcomes in the subset of SHASTA study patients who were treatment naïve were not specifically evaluated.

It is understood that outcomes in RVO are better with earlier treatment [11]. The mean duration of macular edema prior to DEX implant treatment in the SHASTA study was 18.4 months, but patients who were treatment naive were likely to have had a shorter duration of edema. Treatment of newly diagnosed RVO with a long-acting implant has potential to effectively treat edema and restore vision without a need for frequent intravitreal injections of anti-VEGF or corticosteroid. In the present report, subanalysis of treatment-naïve patients in the SHASTA study was undertaken to evaluate outcomes in patients who received DEX implant as initial therapy.

## Methods

A multicenter, retrospective, open-label chart review study was conducted at 26 sites in the United States. The study adhered to the tenets of the Declaration of Helsinki and was conducted in accordance with Health Insurance Portability and Accountability Act regulations. All 26 sites obtained approval of an Institutional Review Board/Ethics Committee (either the New England Institutional Review Board, the Wake Forest University Health Sciences Institutional Review Board, or the UC Davis Institutional Review Board), and all patients provided written informed consent.

The study design and methods have been described in detail previously [10] and are summarized here. Patient inclusion criteria were age 18 years or older, diagnosed with macular edema secondary to RVO in the study eye, received at least 2 injections of DEX implant 0.7 mg in the study eye, and had medical chart data available for at least 3 months after the last DEX implant. The primary exclusion criterion was previous treatment with DEX implant as part of or during a clinical study. Other treatments and procedures for complications of RVO during the study period were allowed. If both eyes were eligible for the study, the eye that had received the largest number of DEX implants was designated as the study eye.

Data were collected from patient records from the time of the first DEX implant (baseline) through at least 3 months and up to 6 months after the last DEX implant. These data included BCVA (Snellen measurements were converted to approximate number of Early Treatment Diabetic Retinopathy Study letters, where 5 letters = 1 line), central retinal thickness by OCT, fluorescein angiography, DEX implant injections and other RVO treatments and procedures, intraocular pressure (IOP), cataract and glaucoma surgeries, and adverse events. Demographics and ophthalmic history were taken from records of the baseline visit. The diagnosis of RVO-related macular edema and the assessments of BCVA, central retinal thickness, and IOP were not standardized, and patient examinations and treatment were at the discretion of the investigator.

Results in the patient subgroup of treatment-naïve patients, who had not been treated previously for complications of RVO, were analyzed as previously described for results in the total patient population and in the patient subgroups diagnosed with BRVO or CRVO [10]. Analyses of BCVA and central retinal thickness were based on observed values and the peak effect of treatment (greatest number of lines read and thinnest central retinal thickness) measured between DEX implant injections. A subanalysis evaluated results in treatment-naïve patients diagnosed with BRVO or CRVO. Mean changes in BCVA from baseline were also evaluated in phakic *versus* pseudophakic eyes.

SAS version 9.2 (SAS Institute Inc., Cary, NC) and a 2-sided alpha level of 0.05 were used for statistical analysis. Changes in BCVA and central retinal thickness from baseline following each DEX implant injection were analyzed using analysis of covariance with fixed effects of subgroup and diagnosis (BRVO or CRVO) and baseline BCVA or central retinal thickness as the covariate in the model. Changes from baseline in BCVA and central retinal thickness were analyzed with paired *t* tests. Categorical variables were compared with Pearson Chi-square or Fisher exact tests.

## Results

Of the 289 patients in the study, 39 (13.5 %) were treatment-naïve. Table 1 summarizes key baseline characteristics of the treatment-naïve patients and study eyes. Eighteen patients were diagnosed with BRVO and 21 with CRVO. The mean duration of RVO-associated macular edema prior to the first DEX implant was 4.9 months, and the macular edema was severe (mean central retinal thickness, 550 μm; range 219–1125) and associated with poor visual acuity (mean BCVA, 20/125 Snellen).

**Table 1:** Baseline demographics of treatment-naïve patients

Characteristic	Treatment-naïve
	(*N* = 39)
Mean (SD) age, years	68.4 (12.6)
Range	39–88
Sex, n (%)	
Female	21 (53.8)
Male	18 (46.2)
Race/Ethnicity, n (%)	
White	20 (51.3)
Black, Asian, or other	3 (7.7)
Not recorded in chart	16 (41.0)
Diagnosis, n (%)	
BRVO	18 (46.2)
CRVO	21 (53.8)
Mean (SD) duration of macular edema, months	4.9 (16.7)
Median	1.2
Range	0 to 105
Prior treatment for complications of retinal vein occlusion, n (%)	0 (0)
Glaucoma or ocular hypertension at baseline, n (%)	
Yes	6 (15.4)
No	23 (59.0)
Not recorded in chart	10 (25.6)
Using IOP-lowering medication at baseline, n (%)	3 (7.7)
History of IOP response to steroid, n (%)	
Yes	1 (2.6)
No	24 (61.5)
Not recorded in chart	14 (35.9)
Lens status, n (%)	
Phakic	23 (59.0)
Pseudophakic	15 (38.5)
Not recorded	1 (2.6)
Mean (SD) BCVA, lines	8.5 (5.1)
Snellen	20/125
Mean (SD) central retinal thickness, μm	550 (207)

*BCVA* best-corrected visual acuity, *BRVO* branch retinal vein occlusion, *CRVO* central retinal vein occlusion, *IOP* intraocular pressure, *SD* standard deviation, *VEGF* vascular endothelial growth factor

### Treatment

Follow-up data after the first DEX implant in treatment-naïve patients were collected for a mean of 13.9 months (standard deviation [SD], 4.6; range 5.3–25.1). The mean number of DEX implants received during the study period was 2.9 (SD, 1.4; range 2–8) and the mean time between DEX implants, calculated per patient, was 177 (SD, 68; range 91–365) days. A total of 20 treatment-naïve patients received only 2 DEX implant treatments; follow-up data were collected for these patients for a mean of 4.6 months after their last DEX implant treatment.

Twelve (30.8 %) treatment-naïve patients received DEX implant and no other treatments for RVO during the study period. The mean time between DEX implants for these patients was 154 (SD, 49) days. The remaining 27 (69.2 %) treatment-naïve patients received DEX implant and adjunctive RVO treatment, most commonly anti-VEGF injections (Table 2).

**Table 2:** Treatments used adjunctively with DEX implant for complications of RVO in treatment-naïve patients

Treatments used in addition to DEX implant	Treatment-naïve patients
	(*N* = 39)
Any treatment, n (%)	27 (69.2)
Intravitreal injection	
Anti-VEGF, n (%)	23 (59.0)
Ranibizumab, n (%)	16 (41.0)
Bevacizumab, n (%)	9 (23.1)
Mean number of ranibizumab or bevacizumab injections in patients receiving anti-VEGF	3.2
Range	1–6
Laser photocoagulation	
Focal, n (%)	11 (28.2)
Panretinal, n (%)	1 (2.6)

*DEX implant* dexamethasone intravitreal implant, *RVO* retinal vein occlusion, *VEGF* vascular endothelial growth factor

In the patients who received DEX implant and adjunctive RVO treatment, the mean time between DEX implants was 187 (SD, 74) days. For the 23 patients who received anti-VEGF treatment as well as DEX implant, the mean time between DEX implants was 195 days (SD, 78; range 91–365). Follow-up data were collected in this subset of patients for a mean of 13.9 months (SD, 3.9; range 6.3–20.1) after the first DEX implant. The anti-VEGF treatment was given at a mean of 3.1 months after the most recent DEX implant; it was administered between DEX implant injections in 17 patients and after the last DEX implant injection in 6 patients. Focal laser treatment was administered in 11 (28.2 %) treatment-naïve patients during the study period, more commonly in BRVO (9/18 patients, 50 %) than CRVO (2/21 patients, 9.5 %) (*P* = 0.005). One treatment-naïve patient with BRVO had panretinal laser photocoagulation during the study period.

The mean (SD) time between DEX implants was 154 (50) days in treatment-naïve patients with BRVO and 197 (77) days in treatment-naïve patients with CRVO. The mean total number of intravitreal injections (DEX implant plus anti-VEGF) received by treatment-naïve patients during the study period was 4.8 (SD, 2.2; range 2–8).

### Efficacy analysis

**Fig. 1:**
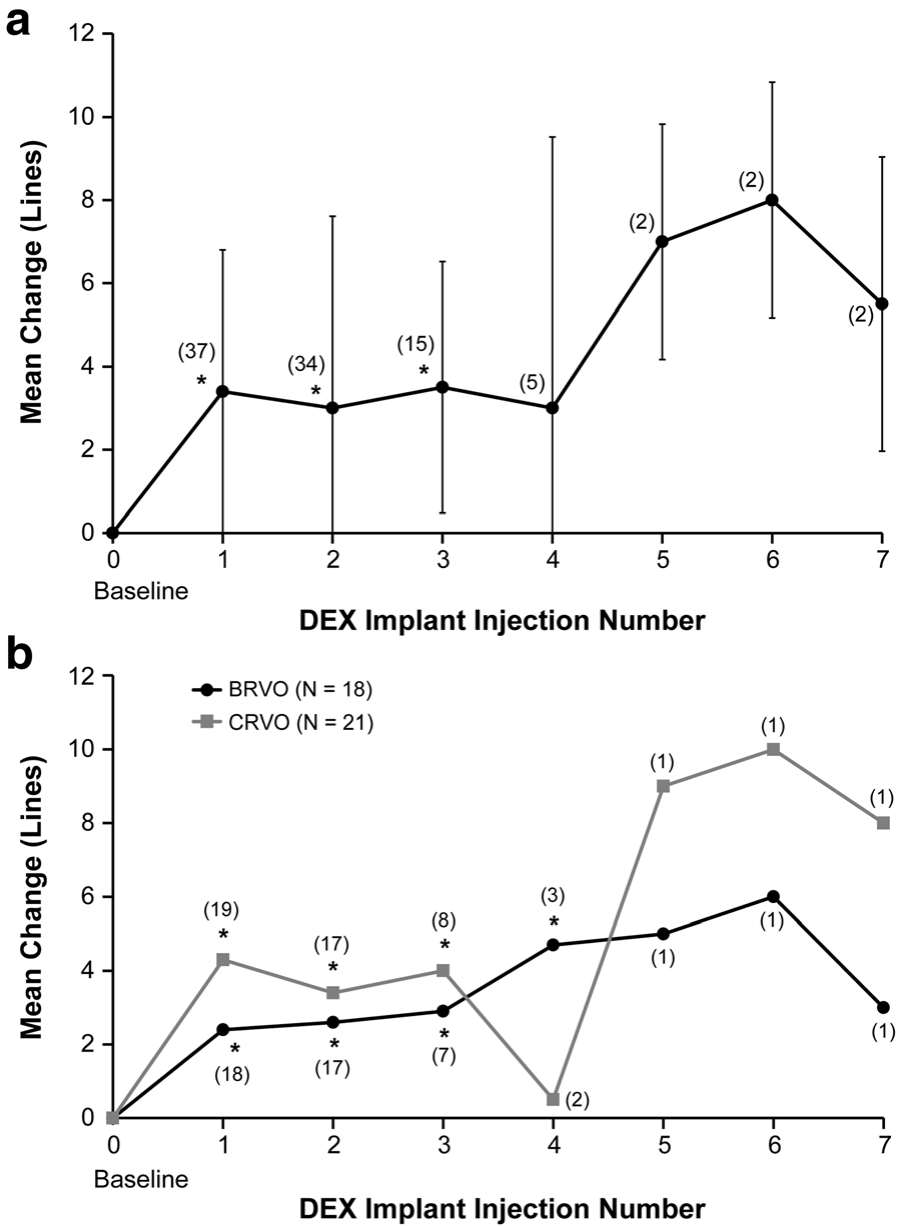
Mean change in best-corrected visual acuity (BCVA) from baseline after each dexamethasone intravitreal implant (DEX implant). Results are shown for patients with retinal vein occlusion who were treatment-naïve at the time of the first DEX implant treatment. **a** All treatment-naïve patients. **b** Treatment-naïve patients diagnosed with branch or central retinal vein occlusion (BRVO, CRVO). Numbers in parentheses indicate number (n) of patients included in analysis (all patients with available data). Error bars, standard deviation. *P ≤ 0.034 vs baseline

**Fig. 2:**
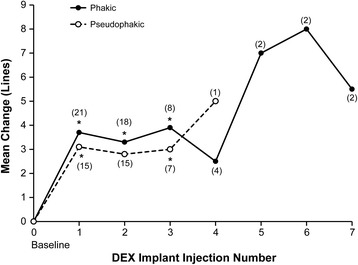
Mean change in best-corrected visual acuity from baseline after each dexamethasone intravitreal implant (DEX implant). Results are shown in phakic and pseudophakic eyes with retinal vein occlusion that were treatment-naïve at the time of the first DEX implant treatment. No pseudophakic eyes received >4 DEX implant treatments. Numbers in parentheses indicate number (n) of patients included in analysis (all patients with available data). *P ≤ 0.020 vs baseline

**Fig. 3:**
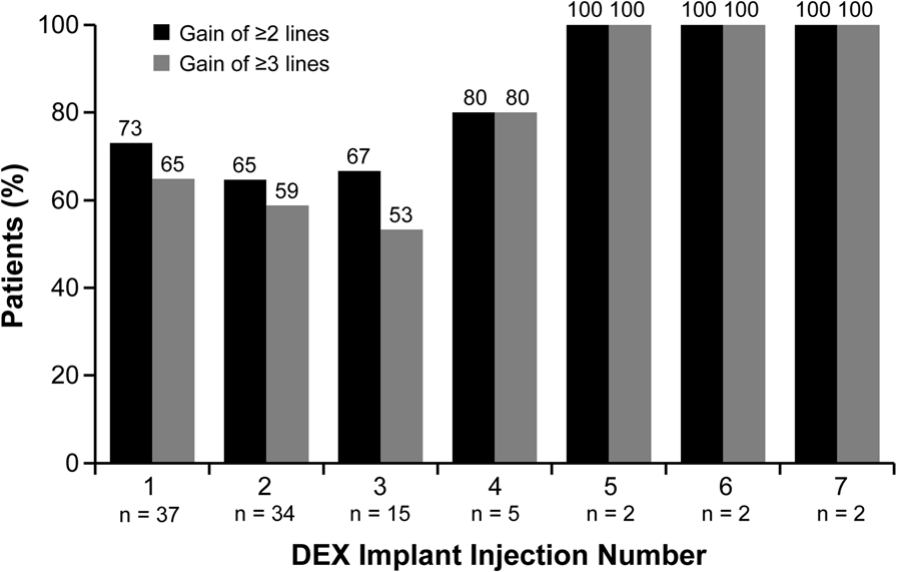
Gain of at least 2 or 3 lines in best-corrected visual acuity. The percentage of treatment-naïve patients with retinal vein occlusion who gained at least 2 or 3 lines in best-corrected visual acuity from baseline after each dexamethasone intravitreal implant (DEX implant) is shown. Numbers in parentheses indicate number (n) of patients included in analysis (all patients with available data)

Mean changes from baseline BCVA after the first through seventh DEX implant injections ranged from +3.0 to +8.0 lines in treatment-naïve patients and were statistically significant after the first through third injections (Fig. 1a). Improvement in BCVA after DEX implant treatment was seen in treatment-naïve patients diagnosed with either BRVO or CRVO (Fig. 1b). Changes in BCVA from baseline after each DEX implant were similar in phakic and pseudophakic eyes (Fig. 2). The percentage of treatment-naïve patients who had at least a 2-line or 3-line gain in BCVA from baseline after the first through seventh DEX implants is shown in Fig. 3. Overall, during the study period, 83.8 % (31/39) of treatment-naïve patients had at least a 2-line improvement in BCVA and 70.3 % (26/39) had at least a 3-line improvement in BCVA.

**Fig. 4:**
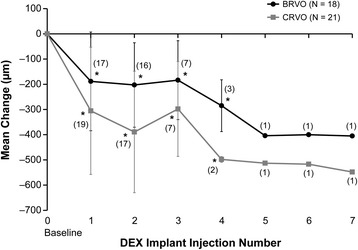
Mean change in central retinal thickness from baseline after each dexamethasone intravitreal implant (DEX implant). Results are shown for patients with retinal vein occlusion who were treatment-naïve at the time of the first DEX implant treatment. Numbers in parentheses indicate number (n) of patients included in analysis (all patients with available data). Error bars, standard deviation. *P ≤ 0.004 vs baseline

Mean decreases from baseline central retinal thickness after the first through seventh DEX implants ranged from 241 μm to 477 μm. Statistically significant decreases from baseline central retinal thickness occurred after the first through fourth DEX implants in treatment-naïve eyes with BRVO, as well as treatment-naïve eyes with CRVO (Fig. 4). During the study period, 56.4 % (22/39) of treatment-naïve patients achieved a central retinal thickness of ≤250 μm. Diagnosis had no significant effect on achievement of a dry macula: 50.0 % (9/18) of treatment-naïve eyes with BRVO and 61.9 % (13/21) of treatment-naïve eyes with CRVO achieved a central retinal thickness of ≤250 μm during the study period (*P* = 0.455).

In additional subset analyses, among the treatment-naïve patients who received only DEX implant and no adjunctive RVO treatment during the study period (*n* = 12), 83.3 % gained at least 2 lines in BCVA during the study period, 75.0 % gained at least 3 lines in BCVA during the study period, and 66.7 % achieved a central retinal thickness of ≤250 μm.

### Safety analysis

As in the total patient population, the most common adverse event associated with DEX implant treatment in the treatment-naïve subgroup was an increase in IOP. During the study period, 12 (30.8 %) treatment-naïve patients had an increase in IOP from baseline of at least 10 mm Hg at one or more measurements (Table 3). The initial ≥10 mm Hg IOP increase measured was after the first DEX implant injection in each of these patients. IOP-lowering medication was used as a consequence of the retina treatment in 30.8 % of treatment-naïve patients. No patients in the treatment-naïve subgroup required laser or incisional glaucoma surgery during the study period.

**Table 3:** Intraocular pressure safety parameters in treatment-naïve patients

Parameter, n (%)	Treatment-naïve patients
	(*N* = 39)
At any time during study	
Increase from baseline ≥10 mm Hg	12 (30.8)
Post-baseline IOP ≥25 mm Hg	15 (38.5)
Post-baseline IOP ≥35 mm Hg	5 (12.8)
At final visit	
Increase from baseline ≥10 mm Hg	4 (10.3)
IOP ≥25 mm Hg	4 (10.3)
IOP ≥35 mm Hg	0 (0)
Glaucoma surgery (laser/incisional)	0 (0)
IOP-lowering medication used secondary to retina treatment	12 (30.8)

*IOP* intraocular pressure

Among the 23 phakic eyes at baseline, 5 (21.7 %) had cataract surgery during the study period. The cataract surgery was performed at a mean of 8.9 months (range, 2.0–16.6) after the first DEX implant. In 4 of the eyes, the lens opacity at baseline prior to DEX implant treatment was grade 2 or 3; lens opacity for the other eye was not recorded in the chart.

## Discussion

This subgroup analysis of the SHASTA study evaluated the safety and efficacy of treatment with 2 or more DEX implants in eyes with RVO-associated macular edema that had received no previous treatment for complications of RVO. Within this subgroup of treatment-naïve patients, changes in BCVA were similar in phakic and pseudophakic study eyes. Each subsequent DEX implant led to substantial improvement in BCVA, regardless of whether the eyes were phakic or pseudophakic.

Treatment options for RVO-associated macular edema include intravitreal corticosteroids, intravitreal anti-VEGF agents, and laser in BRVO. While anti-VEGF treatment provides good efficacy in many patients, frequent injections may be needed and some patients may be partial responders or nonresponders [12]. The clinical trials for regulatory approval of ranibizumab for treatment of macular edema after BRVO or CRVO used monthly injections of ranibizumab [13, 14], which can be burdensome for patients. Therefore, there is a need for initial treatment options other than anti-VEGF. An advantage of DEX implant therapy is that with sustained release of drug, the duration of effectiveness is longer, and injections can be given less frequently, decreasing the burden of therapy. Chart review studies have suggested that in clinical practice, retreatment with DEX implant typically is given 4 to 6 months after the initial treatment [10, 15, 16]. The average interval between DEX implant treatments for treatment-naïve patients in the SHASTA study was 5.8 months.

The adverse events commonly associated with DEX implant—IOP increases and cataract—are expected with intraocular corticosteroid treatment, but occur at lower frequency than with other corticosteroids that could be used off-label [17–19], probably because of differences in the ocular distribution [19] and pharmacological profiles [20] of the corticosteroids. These corticosteroid-related adverse effects are typically managed with IOP-lowering medication and cataract surgery, respectively. Intravitreal anti-VEGF agents are less frequently associated with IOP increases [21] and are not associated with cataract development, and the ocular safety profile of intravitreal anti-VEGF is generally favorable. However, intravitreal anti-VEGF treatment has a potential for serious systemic adverse events, including arterial thromboembolic events [22].

The present study showing substantial improvement in BCVA following DEX implant treatment in treatment-naïve patients is consistent with results of a recent single-center chart review study [15]. In another recent chart review study [23], DEX implant provided greater improvement in BCVA in treatment-naïve patients than in previously treated patients. These results may be due in part to a shorter mean duration of macular edema in treatment-naïve patients, as early treatment of BRVO-associated macular edema with DEX implant is most effective [11].

Anatomic improvements in central retinal thickness were seen following DEX implant treatment in both BRVO and CRVO. Overall, 56.4 % of treatment-naïve patients achieved a central retinal thickness ≤250 μm. We used central retinal thickness ≤250 μm as the criterion for treatment response in our analysis, because this central retinal thickness is considered to be in the normal range when measured with time-domain OCT. However, measurements of central retinal thickness on spectral-domain OCT are larger than on time-domain OCT [24], and the machine used for OCT was not standardized across study sites. Therefore, it is possible that patients could have had measurements done with spectral domain OCT and achieved normal central retinal thickness, but not achieved the 250 μm thickness used in the analysis as the cutoff for treatment response.

This study has the limitations inherent in nonrandomized, observational, chart review studies. There was no standardization of patient evaluations, either in terms of the timing of follow-up or the assessments performed. Data on the OCT machines used were not available, and use of different OCT machines may have affected the central retinal thickness results, resulting in underestimation of the number of patients achieving normal central retinal thickness. Because the period of follow-up after the last DEX implant was 3 to 6 months, there is a possibility that the 4 patients with elevated IOP at the last follow-up may have gone on to need additional treatments or procedures for IOP management. Only 39 patients in the study were treatment-naïve, and only 12 treatment-naïve patients received DEX implant alone *versus* with other treatments. Nonetheless, the results in this small sample suggest that treatment with DEX implant alone can be effective as initial therapy for RVO-associated macular edema.

## Conclusions

This subgroup analysis of the SHASTA study has demonstrated that treatment with 2 or more DEX implants, alone or with other adjunctive RVO treatment, improves visual acuity, reduces central retinal thickness, and has an acceptable safety profile in patients with newly diagnosed and previously untreated RVO-associated macular edema.
